# circGLI3 Inhibits Oxidative Stress by Regulating the miR-339-5p/VEGFA Axis in IPEC-J2 Cells

**DOI:** 10.1155/2021/1086206

**Published:** 2021-08-11

**Authors:** Zhi-xin Li, Li-xue Wang, Yu Zhang, Wei Chen, Yong-qing Zeng

**Affiliations:** Shandong Provincial Key Laboratory of Animal Biotechnology and Disease Control and Prevention, College of Animal Science and Technology, Shandong Agricultural University, No. 61 Daizong Street, Tai'an City, Shandong Province, 271018, China

## Abstract

As a new type of noncoding RNA, circular RNA (circRNA) is stable in cells and not easily degraded. This type of RNA can also competitively bind miRNAs to regulate the expression of their target genes. The role of circRNA in the mechanism of intestinal oxidative stress (OS) in weaned piglets is still unclear. In our research, diquat (DQ) was used to induce OS in small intestinal epithelial cells (IPEC-J2) to construct an OS cell model. Mechanistically, dual luciferase reporter assays, fluorescence in situ hybridization (FISH), and western blotting were performed to confirm that circGLI3 directly sponged miR-339-5p and regulated the expression of VEGFA. Overexpression of circGLI3 promoted IPEC-J2 cell proliferation, increased the proportion of S-phase cells (*P* < 0.01), and reduced reactive oxygen species (ROS) generation when IPEC-J2 cells were subjected to OS. circGLI3 can increase the activity of glutathione peroxidase (GSH-Px) and the total antioxidant capacity (T-AOC) in IPEC-J2 cells and reduce the malondialdehyde (MDA) content and levels of inflammatory factors. Therefore, overexpression of circGLI3 reduced oxidative damage, whereas miR-339-5p mimic counteracted these effects. We identified a regulatory network composed of circGLI3, miR-339-5p, and VEGFA and verified that circGLI3 regulates VEGFA by directly binding miR-339-5p. The expression of VEGFA affects IPEC-J2 cell proliferation, cell cycle progression, and ROS content and changes the levels of antioxidant enzymes and inflammatory factors. This study reveals the molecular mechanism by which circGLI3 inhibits OS in the intestine of piglets and provides a theoretical basis for further research on the effect of OS on intestinal function.

## 1. Introduction

Oxidative stress (OS) is usually defined as a state of imbalance between the production of free radicals or reactive oxygen species (ROS) in the body and the body's antioxidant protection mechanisms [1]. This imbalance leads to damage to important biomolecules and cells, with a potential impact on the whole organism [2]. In addition, oxidative imbalance is correlated with protein modification, lipid oxidation, and nucleic acid cleavage, which may further impair cellular function in piglets [3]. Currently, numerous studies have shown that OS may be the main factor leading to the occurrence and development of many diseases in piglets [4]. Furthermore, it reported that intestinal paracellular permeability and ROS production would increase under the condition of oxidative stress in the small intestine [5, 6]. Besides, high-level oxidative stress induced by ROS is reported to regulate the paracellular permeability and tight junction opening of epithelial cells [7, 8]. Interestingly, once the occurrence of oxidative stress in the intestinal mucosa was examined, there is an increase in ROS production and the activity of myeloperoxidase, accompanied by the lipid peroxidation that occurred [9]. These evidences showed that small intestine cells might be sensitive to OS.

Noncoding RNA (ncRNA), which comprises various RNA types, including microRNAs (miRNAs), long ncRNAs (lncRNAs), and circular RNAs (circRNAs), has emerged as a powerful gene expression regulator that plays crucial roles in human intestinal function [10]. circRNAs do not have a 5′ end cap or a 3′ end poly(A) tail and form a closed loop structure through covalent bonding [11]. In recent years, the biological function of circRNAs has become a hotspot in scientific research. In particular, circRNAs are involved in posttranscriptional regulation and may act as miRNA sponges or competitive endogenous RNAs (ceRNAs) to regulate the expression of target genes [12]. Evidence showed that circRNAs were demonstrated to be abundant, stable, and conserved as competing endogenous RNAs (Jeck et al. 2013). In addition, studies have shown that some circRNAs are regulated by OS and promote ROS-induced cell apoptosis, inflammation, and disease [13, 14]. miRNAs are a group of small, noncoding, single-stranded RNAs that play critical roles in many biological processes and molecular functions [15]. The functional definition of miRNA primarily involves translation inhibition and degradation of specific target genes to achieve inhibition of protein synthesis [16, 17]. For ceRNA research, most circRNAs function as miRNA cavernous bodies and operate by inhibiting the activity of target genes. However, whether circRNAs and miRNAs participate in OS regulation in the intestine in piglets is not yet clear.

Here, based on previous RNA-seq results, we first identified a novel circRNA derived from the GLI3 gene in the jejunum mucosa of piglets. Subsequently, to obtain insight into the function and underlying molecular mechanism of circGLI3 in OS development and progression, circGLI3 upregulation was induced in IPEC-J2 cells using diquat (DQ). The results show that circGLI3 can act as a sponge of miR-339-5p to affect the expression of VEGFA and eventually modulate IPEC-J2 cell proliferation and ROS content. Collectively, our findings provide new insights into the mechanism of intestinal OS.

## 2. Materials and Methods

### 2.1. Cell Lines and Treatment

Intestinal porcine epithelial cells (IPEC-J2) were purchased from Beijing BeiNa Chuanglian Biological (BNCC) Technology Research Institute. After the cells were received, they were stabilized in the incubator for approximately 4 hours, and then, the medium for culture or passage was adjusted according to the cell density. The IPEC-J2 cell line was cultured in a humidified incubator containing 5% CO_2_ at 37°C. The growth conditions required for cell growth were as follows: 89% high-glucose DMEM+10% FBS+1% penicillin and 5% CO_2_+95% air. The cryopreservation conditions were as follows: 50% medium+40% serum+10% DMSO.

Before treatment, confluent monolayers of IPEC-J2 cells were washed twice with a plain medium. IPEC-J2 cells were treated with DQ (Sigma-Aldrich, Saint Louis, USA) solution, which was freshly prepared prior to each experiment. After incubation with DQ for 24 hours, the cells were washed twice with plain medium before being subjected to subsequent procedures.

### 2.2. RNA Extraction and Quantitative Real-Time Polymerase Reaction (qRT-PCR)

Total RNA was extracted from stably transfected IPEC-J2 cells and corresponding normal tissues/cells using TRIzol reagent (Takara, Japan), and genomic DNA (gDNA) was isolated with a FastPure DNA Isolation kit (Vazyme, China) according to the manufacturer's instructions. Primers were designed with Primer3 software (version 4.1.0) and synthesized by Sangon Biotech (Shanghai, China). SYBR® Premix ExTaqTM (Takara, Dalian, China) was used for quantitative real-time polymerase chain reactions in a LightCycler® 96 Real-Time PCR System (Roche). The reaction mixture consisted of 1 *μ*L of cDNA, 0.5 *μ*L of forward primer, 0.5 *μ*L of reverse primer, 10 *μ*L of SYBR premix, and 10 *μ*L of double-distilled water. The cycling conditions were predenaturation at 94°C for 10 min, followed by 45 cycles of denaturation at 94°C for 15 s, annealing at 60°C for 60 s, and extension at 72°C for 10 min. GAPDH was used as the control, and the 2^−*ΔΔ*CT^ method was used to calculate the relative expression of circRNAs and mRNAs.

### 2.3. Vector Construction and Stable Transfection

To construct a stable overexpression circGLI3 vector, full-length circGLI3 cDNA was synthesized and cloned into the overexpression vector PLCDH-circ, which contained a front and back circular frame to promote RNA circularization. The empty vector without the circGLI3 sequence was used as a negative control. The miR-339-5p mimic, inhibitor, and negative control mimic were synthesized by GenePharma (Shanghai, China). The wild-type and mutant VEGFA sequences were synthesized and subcloned into the polyclonal site of a psiCHECK 2.0 vector, and the constructed vectors were named VEGFA-WT and VEGFA-MUT. Stable transfection of IPEC-J2 cells was performed as previously described.

### 2.4. Luciferase Reporter Assay

The VEGFA 3′UTR sequence and the corresponding mutated sequence were designed, synthesized, and inserted into the luciferase reporter vector psiCHECK-2 (Gene-Pharma, China), and the obtained constructs were termed VEGFA-3′UTR-WT and VEGFA-3′UTR-MUT. IPEC-J2 cells were transfected with plasmids encoding miR-339-5p mimic or inhibitor. Then, the relative luciferase activity was examined using a dual luciferase assay kit (Promega, USA) in line with the manufacturer's protocol.

### 2.5. Fluorescence in Situ Hybridization (FISH)

FISH assays were executed to observe the location of circGLI3 and miR-339-5p in IPEC-J2 cells. Cy3-labeled circGLI3 and FAM-labeled miR-339-5p probes were designed and synthesized by GenePharma (Shanghai, China). Hybridization was performed overnight with the GLI3 and miR-339-5p probes according to the manufacturer's instructions.

### 2.6. Cell Counting Kit-8 Proliferation Assay

Before the experiment, normally cultured IPEC-J2 cells were aspirated with the original culture medium, washed with PBS, and digested with trypsin for 3-5 min. The digestion was stopped, and the mixture was pipetted into a single well; a total of 20 *μ*L of the cell suspension was added to 20 *μ*L of Trypan blue and counted. The remaining cell suspension was centrifuged at 1000 rpm for 5 min, resuspended in culture medium, and plated at 12000 cells/well (96-well plate). After overnight adherence, transfection and DQ treatment were performed. Then, 10 *μ*L of CCK-8 solution was added to each well of the 96-well plate. After 4 hours of incubation, stop solution was added, and the OD value was detected with a microplate reader (BioTek USA) at 450 nm.

### 2.7. ROS Content Determination

DQ treatment and cell transfection with plasmids were carried out as described in subsection 2.1 and 2.3. Then, DAPI was added dropwise and incubated with the cells for 5 min in the dark to stain the nuclei, and excess DAPI was removed by washing with PBST 4 times for 5 min each time; the liquid on the cell slide was absorbed with absorbent paper, and the slide was mounted with an antifluorescence quencher. The slides were mounted in liquid, and then, images were obtained under a fluorescence confocal microscope.

### 2.8. Western Blotting

Protein extraction was performed using a RIPA buffer supplemented with protease inhibitor. The protein concentration was determined using a BCA assay kit (Thermo Scientific, USA). Protein samples were separated by 12% SDS-PAGE and then transferred onto PVDF membranes (Millipore, USA). The membranes were stained using an enhanced chemiluminescence (ECL) horseradish peroxidase (HRP) substrate (Bioworld Technology, China) according to the manufacturer's instructions. Images were acquired using a GBOX ChemiXRQ System (Syngene, UK), and protein expression levels were quantified with ImageJ software. The main reagents used included rabbit anti-VEGFA polyclonal antibody (bs-1313R, Bioss), anti-GAPDH antibody (Proteintech, China), ECL (K-12043-D10, China), TRIS (T8060, Solarbio), HRP-labeled goat anti-rabbit IgG (SA00001-2, Proteintech), and HRP-labeled goat anti-mouse IgG (SA00001-1, Proteintech).

### 2.9. Statistical Analysis

Each experiment was conducted in triplicate, and all data were analyzed using SPSS 19.0 and Prism 8 software. Statistically significant differences were set as *P* < 0.05, and one-way analysis of variance and an independent sample *t*-test were used to determine that the differences between groups were statistically significant. All data are expressed as the mean ± standard deviation (^∗^*P* < 0.05, ^∗∗^*P* < 0.01, and ^∗∗∗^*P* < 0.001).

## 3. Results

### 3.1. DQ Suppressed Cell Viability and Changed Cell Morphology

**Figure 1:**
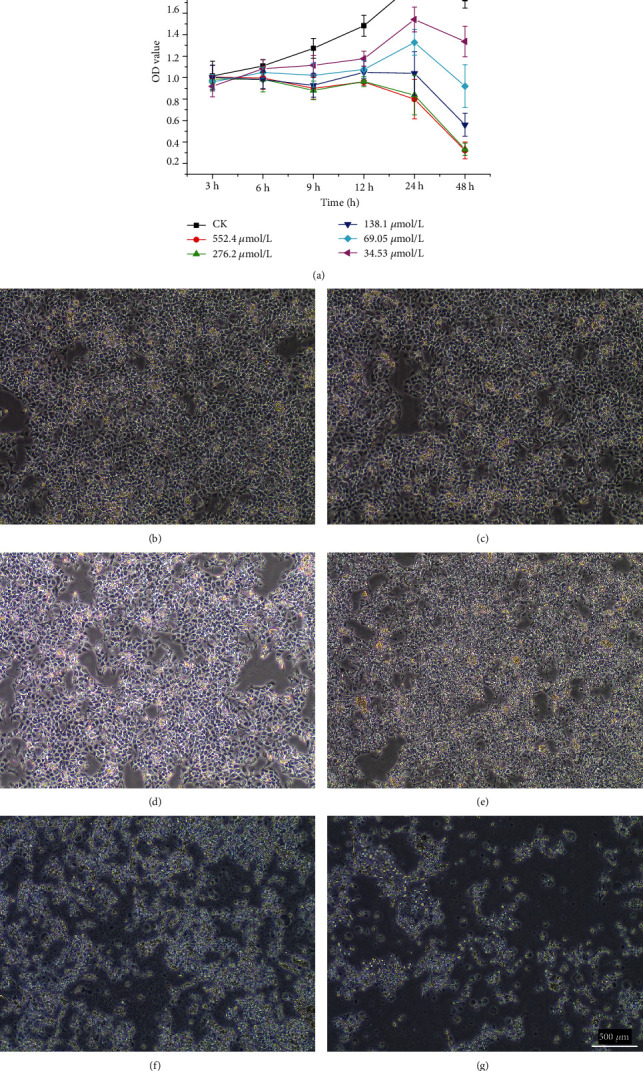
Cell model of OS. The effect of different concentrations of DQ on the viability of IPEC-J2 cells (a) and the effect of DQ on the morphology of IPEC-J2 cells (b–g) (*μ*m).

To investigate the effect of DQ on cell viability and morphology, the cell survival rate was measured with a microplate reader after addition of CCK-8 solution, and the cell morphology was synchronously observed. IPEC-J2 cells were treated with different concentrations of DQ (34.53, 69.05, 138.1, 276.2, and 552.4 *μ*mol/L), and the optical density (OD) was measured at different times (Figure 1(a)). The results showed that the viability of IPEC-J2 cells in the DQ treatment groups was decreased compared with that in the control group. As the DQ concentration increased, the cell viability also gradually and significantly decreased. After 24 hours of DQ treatment of IPEC-J2 cells, the IC50 value was 250.7 *μ*mol/L. After 24 hours of treatment, the cells in the control group grew normally adherent; however, cells in the treatment group became loose and detached and began to float. As the DQ concentration increased, the cell morphology began to change from a diamond shape to a round shape, and cells separated from the surrounding cells. The results showed that DQ treatment destroyed the cell structure and growth of IPEC-J2 cells, which indicated that the integrity and permeability of the intestine were destroyed by oxidative stress (Figures 1(b)–1(g)).

### 3.2. circGLI3 Promoted IPEC-J2 Cell Proliferation and Cell Cycle Progression

**Figure 2:**
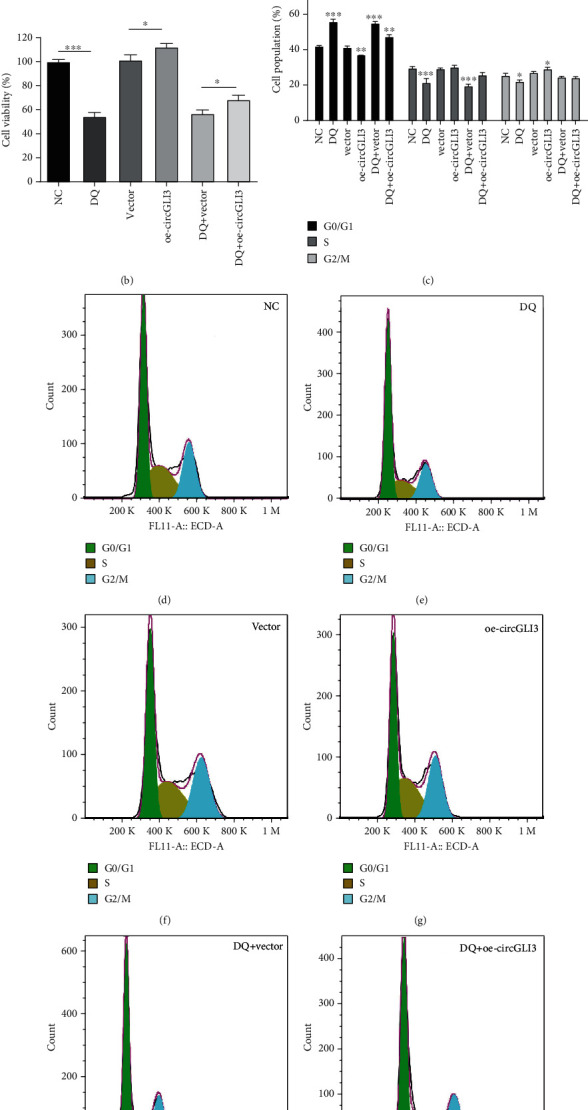
circGLI3 promoted IPEC-J2 cell proliferation and cell cycle progression. The vector of overexpression of circGLI3 was successfully constructed (a). Cell viability (b) and cell cycle distribution (c–i) were assessed in IPEC-J2 cells transfected with oe-circGLI3 (overexpression of circGLI3) or vector (empty PLCDH-circ vector).

To explore the biological function of circGLI3 in IPEC-J2 cells, cell proliferation was measured using CCK-8 assays, and the cell cycle distribution of IPEC-J2 cells was detected via fluorescence-activated cell sorting (FACS) technology. The results demonstrated that increased expression of circGLI3 significantly enhanced cell proliferation and viability, whereas DQ treatment exerted the opposite effects. After transfection with the circGLI3 overexpression vector, the expression of circGLI3 was significantly upregulated, which the successful overexpression of circGLI3 (Figure 2(a)). The increase in circGLI3 expression significantly upregulated the inhibitory effect of DQ on cell proliferation (Figure 2(b)). On the other hand, increased circGLI3 expression significantly reduced the ratio of cells in G0/G1 phase, whereas DQ treatment significantly increased the ratio of cells in G0/G1 phase. Moreover, the proportion of S phase cells in the DQ treatment group was extremely significantly reduced (*P* < 0.001), while the proportion of S phase cells among the cells transfected with the circGLI3 overexpression vector was significantly increased. The results indicate that increased circGLI3 expression can relieve the cell cycle arrest caused by DQ and promote synthesis in the S phase cells (Figures 2(c)–2(i)).

### 3.3. circGLI3 Promoted the Antioxidant System in IPEC-J2 Cells

**Figure 3:**
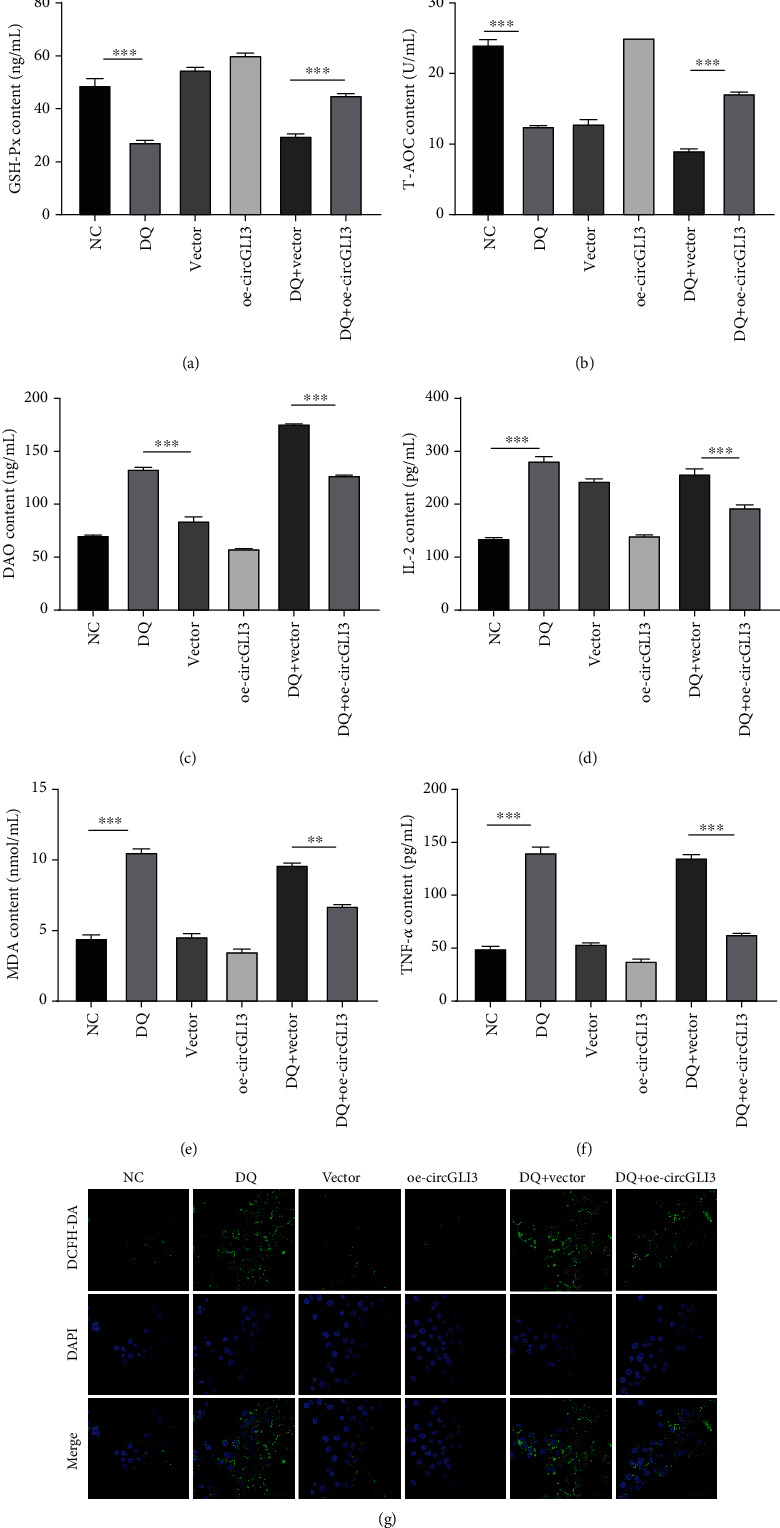
circGLI3 promoted the antioxidant system in IPEC-J2 cells. The GSH-Px (a) and DAO (c) activities, the T-AOC (b) and the IL-2 (d), MDA (e), and TNF-*α* (f) contents were assessed via ELISA. ROS production in IPEC-J2 cells was detected (g).

To verify the effect of circGLI3 on intracellular antioxidant enzyme and ROS levels in IPEC-J2 cells, the activities of glutathione peroxidase (GSH-Px) and diamine oxidase (DAO), total antioxidant capacity (T-AOC), and the malondialdehyde (MDA), IL-2, and TNF-*α* contents were measured using enzyme-linked immunosorbent assays (ELISAs). After treatment with DQ, the GSH-Px activity and T-AOC were slightly decreased, but they started to become significantly upregulated when circGLI3 expression was increased (Figures 3(a) and 3(b)). On the other hand, the MDA, IL-2, TNF-*α*, and DAO contents increased under OS conditions. However, increased expression of circGLI3 lowered their contents (Figures 3(c)–3(f)). The fluorescence value of DCFH-DA is proportional to the ROS level in the cell. After DQ treatment, the ROS level increased significantly. However, increased expression of circGLI3 reduced the ROS level. These results indicate that circGLI3 acted as a negative regulator of OS and an inhibitor of ROS overproduction induced by DQ (Figure 3(g), Supplementary 1).

### 3.4. miR-339-5p Suppressed IPEC-J2 Cell Proliferation and Cell Cycle Progression

**Figure 4:**
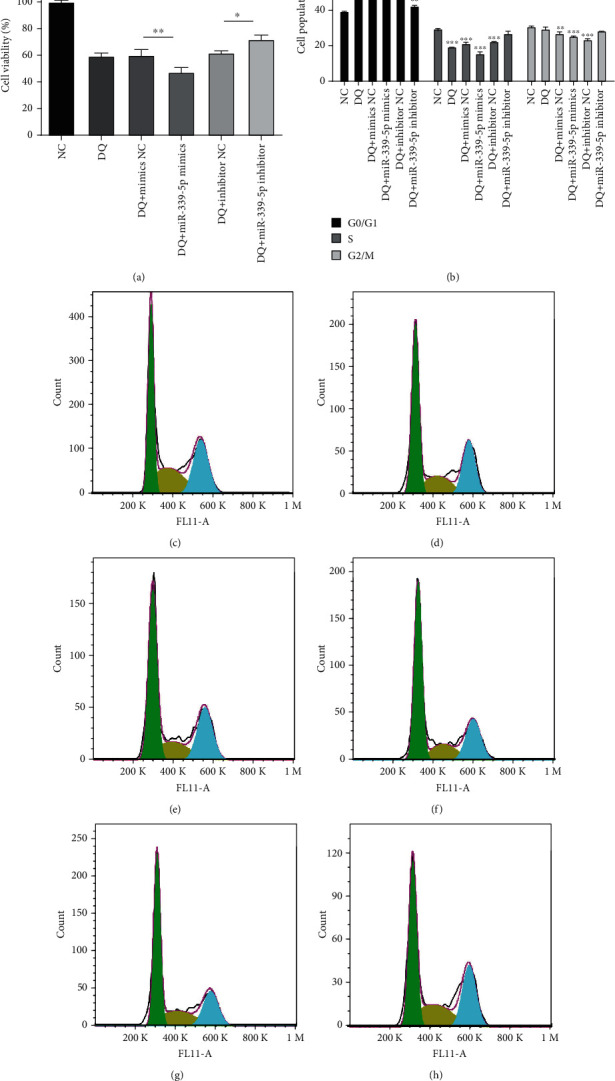
miR-339-5p suppressed IPEC-J2 cell proliferation and cell cycle progression. Cell viability (a) and cell cycle (b–h) were assessed in IPEC-J2 cells transfected with miR-339-5p mimic, inhibitor, mimic NC, or inhibitor NC.

To explore the biological function of miR-339-5p in IPEC-J2 cells, cell proliferation was measured using CCK-8 assays, and the cell cycle distribution of IPEC-J2 cells was detected via FACS technology. The results demonstrated that increased expression of miR-339-5p decreased cell proliferation and viability, whereas decreased miR-339-5p expression exerted the opposite effects. Compared with the control group, DQ treatment significantly reduced the proliferation of IPEC-J2 cells (Figure 4(a)). On the other hand, the proportion of cells in the G0/G1 phase increased significantly after DQ treatment. Increased miR-339-5p expression significantly upregulated the proportion of cells in the G0/G1 phase. However, decreased expression of miR-339-5p significantly reduced the ratio of cells in the G0/G1 phase. The results suggest that miR-339-5p inhibited the proliferation of IPEC-J2 cells (Figures 4(b)–4(h)).

### 3.5. miR-339-5p Suppressed the Antioxidant System in IPEC-J2 Cells

**Figure 5:**
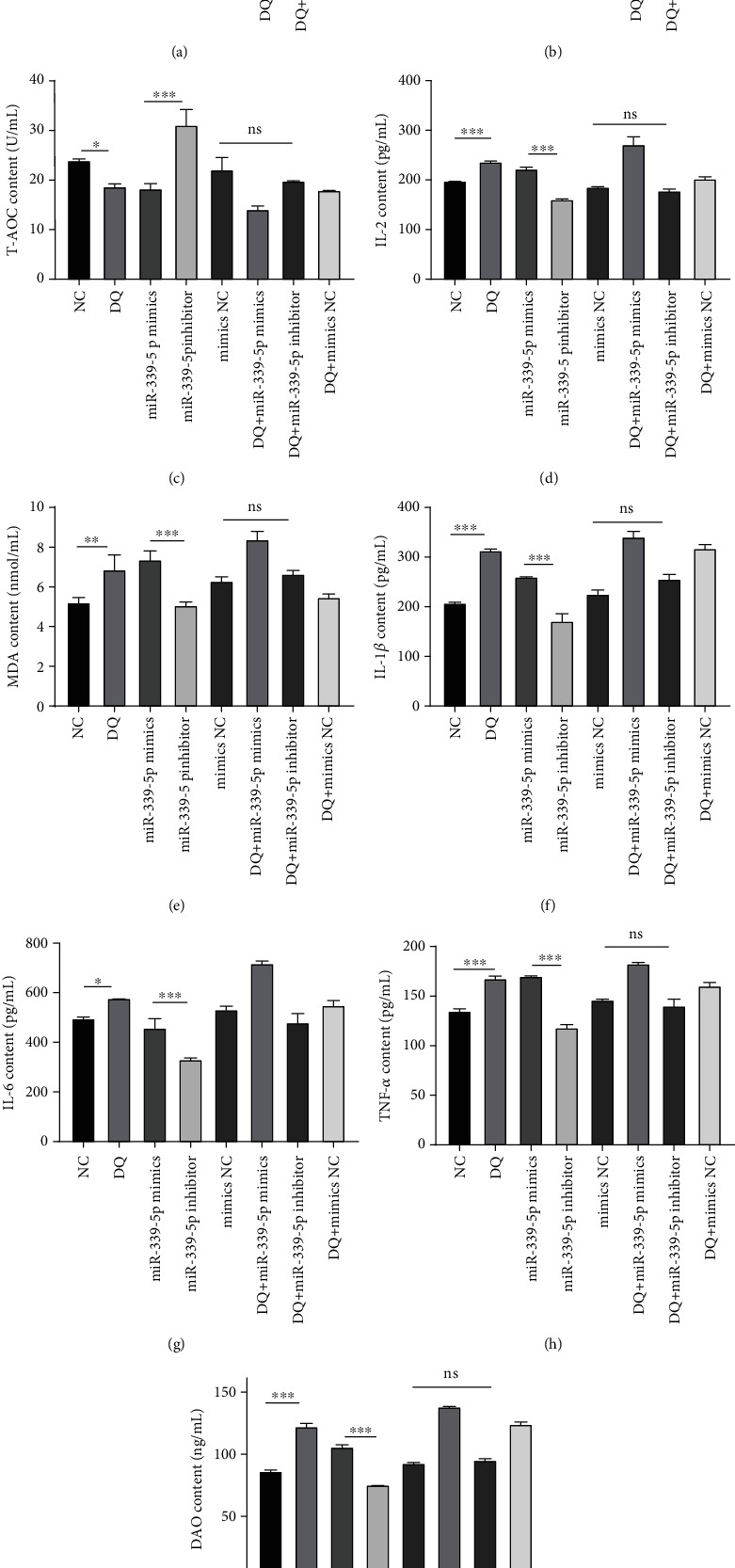
miR-339-5p suppressed the activity of antioxidant enzymes. The activity of SOD (a), GSH-Px (b), and DAO (i); the T-AOC (c); and the IL-2 (d), MDA (e), IL-1*β* (f), IL-6 (g), and TNF-*α* (h) contents were assessed via ELISA.

To explore the effect of miR-339-5p on the IPEC-J2 cell antioxidant system, the activities of superoxide dismutase (SOD) and GSH-Px, T-AOC, and IL-2, MDA, IL-1*β*, IL-6, TNF-*α*, and DAO contents in each group were detected via ELISA. After DQ treatment, the SOD and GSH-Px activities and the T-AOC decreased significantly, but they started to become significantly upregulated when miR-339-5p expression was decreased (Figures 5(a)–5(c)). Moreover, the MDA, IL-2, IL-1*β*, IL-6, TNF-*α*, and DAO contents increased under OS conditions, and increased miR-339-5p expression continued to increase their contents. However, decreased expression of miR-339-5p lowered their contents (Figures 5(d)–5(i)). These results indicate that regular miR-339-5p expression disrupted the antioxidant system and increased the level of inflammation.

### 3.6. circGLI3 Was Mainly Located in the Cytoplasm

**Figure 6:**
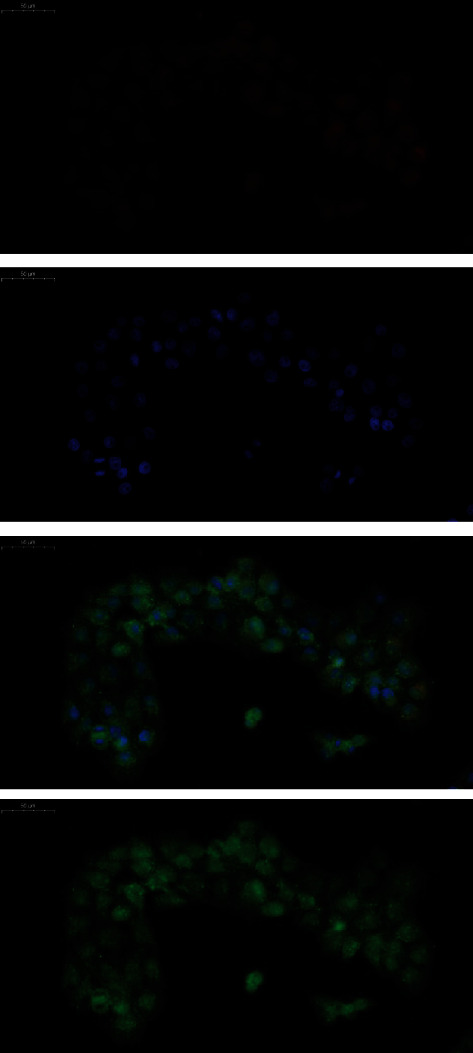
FISH assays were performed to observe the cellular location of circGLI3 and miR-339-5p. Nuclei were stained with DAPI (blue), circGLI3 was labeled with red fluorescence, and miR-339-5p was labeled with green fluorescence.

To explore the region of circGLI3 function in IPEC-J2 cells, a fluorescence in situ hybridization (FISH) assay was performed to observe subcellular colocalization of circGLI3 and miR-339-5p. In our study, most circGLI3 (red) and miR-339-5p (green) were colocalized in the cytoplasm (Figure 6, Supplementary 2).

### 3.7. VEGFA Is a miR-339-5p Target Gene

**Figure 7:**
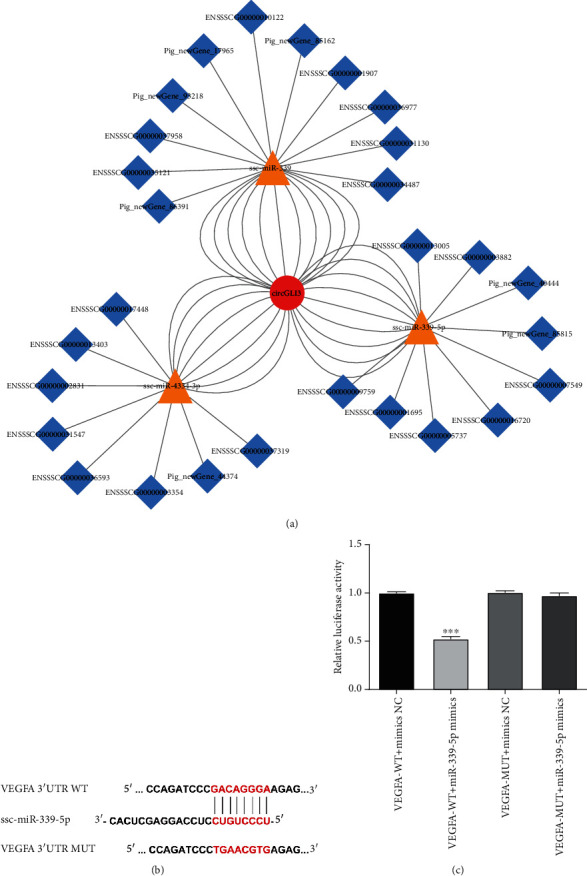
miR-339-5p and VEGFA interaction analysis. (a) The Cytoscape map of circGLI3 network. (b) VEGFA-WT and VEGFA-MUT vectors were constructed according to the binding sites. (c) Relative luciferase activities were detected in IPEC-J2 cells after cotransfection with VEGFA-WT or VEGFA-MUT and mimic or NC.

In previous studies, we verified a binding relationship between circGLI3 and miR-339-5p. The map of the circGLI3 network was shown in Figure 7(a). TargetScan analysis revealed that the 3′UTR of VEGFA contains a miR-339-5p binding site. To verify the binding of miR-339-5p and its target gene, a VEGFA dual luciferase reporter gene vector was constructed by comparing the gene sequence of miR-339-5p and the 3′ end of VEGFA (Figure 7(b)). Then, IPEC-J2 cells were cotransfected with the VEGFA dual luciferase reporter gene vector and miR-339-5p mimic. The luciferase reporter assay showed that miR-339-5p could significantly reduce luciferase activity, indicating specific binding between miR-339-5p and VEGFA (Figure 7(c)).

### 3.8. circGLI3 Served as a miRNA Sponge of miR-339-5p to Regulate VEGFA Expression

**Figure 8:**
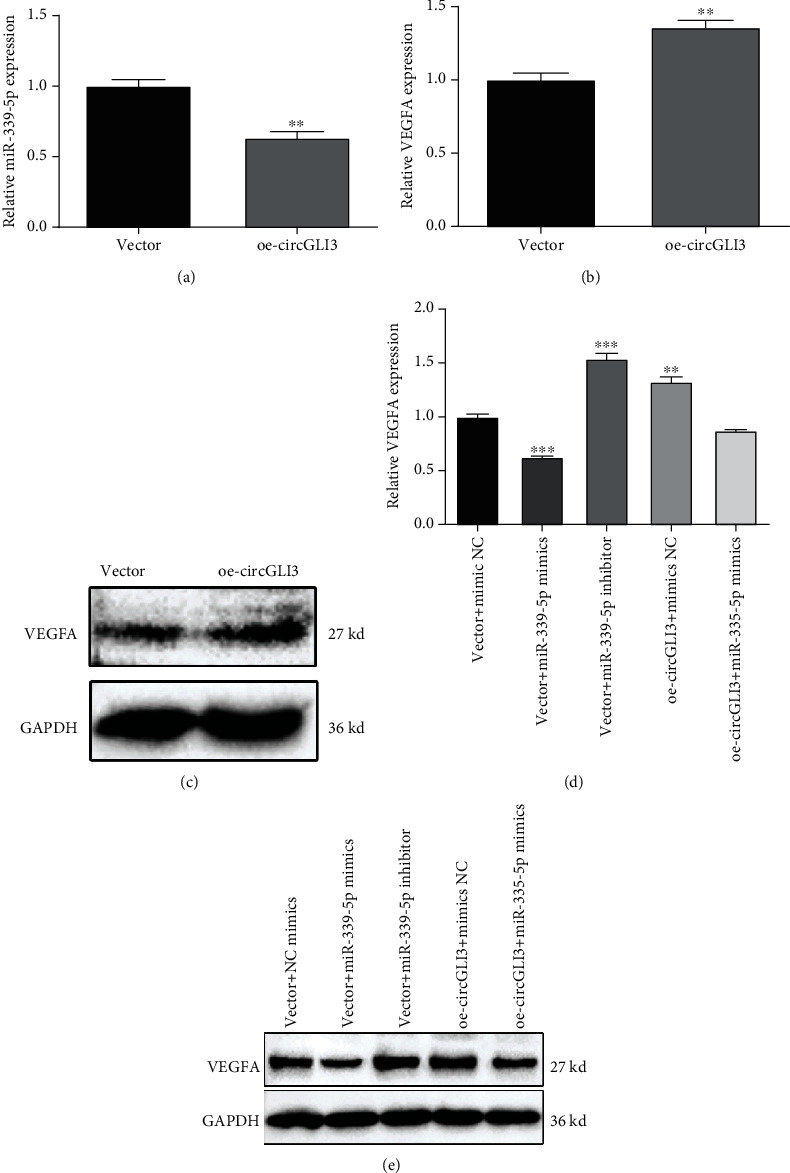
circGLI3 serves as a miRNA sponge of miR-339-5p to regulate VEGFA expression. Relative miR-339-5p (a) and VEGFA (b) expressions and the VEGFA protein level (c) were detected in IPEC-J2 cells transfected with oe-circGLI3 or the vector. Relative VEGFA mRNA (d) and protein levels (e) were detected in IPEC-J2 cells after cotransfection with mimic NC, miR-339-5p mimic, or miR-339-5p inhibitor and oe-circGLI3 or the vector.

To explore the specific mechanism of circGLI3, miR-339-5p, and VEGFA, we performed a series of experiments. After transfection with the circGLI3 overexpression vector, the expression of miR-339-5p was significantly downregulated (Figure 8(a)). Then, qRT-PCR and western blotting assays demonstrated that overexpression of circGLI3 significantly increased the VEGFA mRNA and protein levels (Figures 8(b) and 8(c)). Moreover, overexpression of circGLI3 significantly increased the VEGFA mRNA and protein levels, and miR-339-5p mimic had the opposite effect, whereas cotransfection with oe-circGLI3 and miR-339-5p mimic counteracted these effects in IPEC-J2 cells (Figures 8(d) and 8(e)). These results indicate that circGLI3 may negatively regulate miR-339-5p expression and positively regulate VEGFA expression, further confirming the regulatory relationship between circGLI3 and VEGFA.

### 3.9. circGLI3 Promoted IPEC-J2 Cell Proliferation, Recycling, and Cellular ROS Production through the circGLI3/miR-339-5p/VEGFA Axis

**Figure 9:**
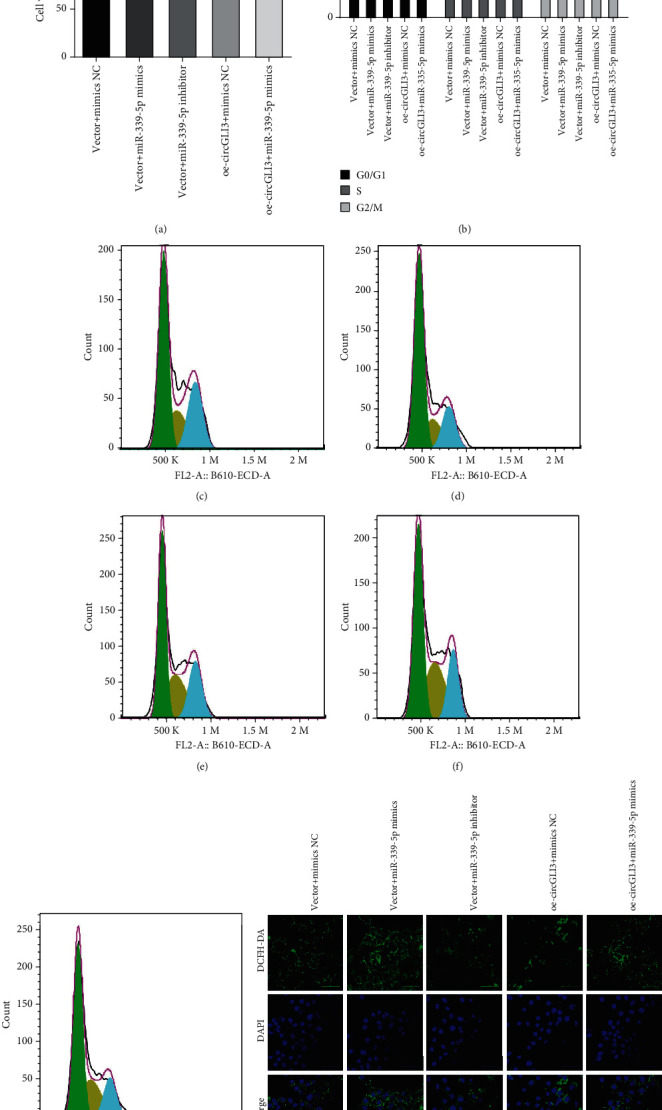
circGLI3 promoted IPEC-J2 cell proliferation, recycling, and cellular ROS production through the circGLI3/miR-339-5p/VEGFA axis. Cell viability (a), cell cycle (b–g), and ROS content (h) were assessed in IPEC-J2 cells after cotransfection with mimic NC, miR-339-5p mimic, miR-339-5p inhibitor, oe-circGLI3, and the vector.

To verify whether circGLI3 exerts its regulatory function through the circGLI3/miR-339-5p/VEGFA axis, rescue experiments were designed using a miR-339-5p inhibitor and mimic. The results showed that miR-339-5p suppressed cell proliferation, whereas circGLI3 overexpression counteracted the suppressive effects induced by miR-339-5p mimic in IPEC-J2 cells (Figure 9(a)). Similarly, miR-339-5p mimic increased the number of cells in the G0/G1 phase; however, cotransfection of IPEC-J2 cells with oe-circGLI3 and miR-339-5p mimic counteracted the effect (Figures 9(b)–9(g)). Furthermore, miR-339-5p mimic increased the intracellular ROS level in IPEC-J2 cells, whereas overexpression of circGLI3 counteracted this effect in IPEC-J2 cells (Figure 9(h)). Collectively, these data indicate that circGLI3 serves as a ceRNA for miR-339-5p to regulate VEGFA expression, thus regulating OS development in IPEC-J2 cells.

## 4. Discussion

IPEC-J2 cells were isolated from the jejunal epithelium of newborn nonlactating piglets [18]. Previous studies have confirmed that IPEC-J2 cells can produce IL-8 under the action of H_2_O_2_ [19]. IPEC-J2 cells are widely used in probiotic screening [20], mycotoxin research [21], intestinal immunity, and inflammation research [22, 23]. DQ is a widely used agricultural chemical, and its toxicity can cause lung, liver, kidney, brain, and other tissue damage [24, 25]. After DQ is injected into an organism, O_2_ is converted into superoxide anions, and various types of ROS are produced through the antioxidant system in the organism [26]. Currently, DQ is a commonly used bipyridyl herbicide and potent prooxidant, which has been used as a model chemical for *in vivo* studies of oxidative stress [27]. Generally, DQ utilizes molecular oxygen to produce superoxide anion radical and subsequently hydrogen peroxide. Further, it induces oxidative stress in animals largely compared to oxidized fish oil [28]. Therefore, DQ has been widely used in OS research as an inducer of cellular OS [29]. The regulatory functions of different circRNAs are not the same. Current research on circRNAs suggests that most circRNAs function as miRNA sponges and function by inhibiting the activity of target genes [30]. In recent years, an increasing number of studies have shown that circRNAs are involved in multiple cell biological processes, such as autophagy, cell proliferation, and apoptosis [31, 32]. In our study, circGLI3 was involved in improving IPEC-J2 cell proliferation, regulation of intracellular antioxidant enzyme activity, and cellular ROS generation, indicating that circGLI3 may play a key role in protecting cells from oxidative damage during DQ-induced OS.

In this study, the MDA content decreased after inhibition of miR-339-5p expression; similarly, the levels of inflammatory factors (IL-2, IL-6, IL-8, and IL-1*β*) decreased significantly. Therefore, miR-339-5p may inhibit the oxidation-reduction imbalance in IPEC-J2 cells by reducing the activities of SOD and GSH-Px and increasing intestinal permeability by increasing the TNF-*α* and DAO contents. Previous evidence has shown that miR-339-5p is a tumor suppressor [33], a regulator of the P53 signaling pathway [34], and can regulate the growth, colony formation, and metastasis of colorectal cancer cells by targeting PRL-1 [35]. In addition, studies have shown that miR-339-5p inhibits the expression of inflammatory factors by targeting specific NF-*κ*B activators [36], and the NF-*κ*B pathway can be activated by external cell stimuli. miR-339-5p plays a key role in the process of inflammation and immune reaction. TNF-*α*, IL-1*β*, IL-2, IL-6, and IL-8 can be used as activating factors of NF-*κ*B [37] and are closely related to OS [38].

VEGFA plays an important role in controlling cell survival, growth, differentiation, adhesion, migration, tumor angiogenesis, wound healing, and tissue repair [39]. Modern medicine has found that VEGFA is involved in many angiogenesis-dependent diseases, including cancer and certain types of inflammatory diabetic retinopathy. Inhibiting VEGFA gene expression leads to an increase in the ROS level in spinal cord cells and activates the *β*-catenin signaling pathway [40]. VEGFA silencing can increase bevacizumab-induced oxidative damage, cardiomyocyte apoptosis, and the ROS level and can change related apoptotic proteins [41]. Moreover, there is evidence that showed that VEGF effects on epithelial cell migration play an important part in epithelial cell restitution by maintaining mucosal homeostasis after mucosal injury [42]. Furthermore, VEGFA is present in intestinal epithelial cells, the lamina propria, and intermuscular plexus where it is involved in positively regulating intestinal cell migration [43]. Besides, the expression of VEGFA is significantly upregulated in colorectal cancer cell lines [44], whereas its expression level in necrotizing enteritis is reported to be significantly reduced.

Considering that circRNA acts as a miRNA sponge in the cytoplasm, we performed FISH experiments in IPEC-J2 cells and observed the subcellular colocalization of circGLI3 and miR-339-5p. The results showed that most circGLI3 (red) and miR-339-5p (green) were located in the cytoplasm. A previous study showed that a direct interaction exists between circGLI3 and miR-339-5p. The dual luciferase reporter assay results suggested that VEGFA is a target gene of miR-339-5p. OS caused by DQ can inhibit IPEC-J2 cell proliferation, hinder cell cycle progression, and increase the intracellular ROS content, thereby aggravating OS-induced damage to cells. To verify whether circGLI3 exerts its regulatory function through circGLI3/miR-339-5p/VEGFA, we designed miR-339-5p inhibitor and mimic rescue experiments. The results verified that overexpression of circGLI3 can increase the VEGFA mRNA and protein levels in IPEC-J2 cells. At the same time, the effects of circGLI3 overexpression were also reversed by miR-339-5p mimic. The results showed that miR-339-5p mimic reversed DQ-induced proliferation, cell cycle progression, and ROS production in IPEC-J2 cells overexpressing circGLI3. In summary, these data indicate that circGLI3 acts as a ceRNA of miR-339-5p to regulate VEGFA expression, thereby participating in OS progression. Given the above evidence, our results suggest the importance of circGLI3 as a novel biomarker of OS in the intestine.

## 5. Conclusions

In this study, IPEC-J2 cells were used to construct a DQ-induced cellular OS model and to predict and verify the ceRNA regulatory network involving circGLI3. circGLI3 can bind its target miR-339-5p in the cytoplasm, upregulate VEGFA mRNA and protein expression, enhance antioxidant enzyme activity, promote IPEC-J2 cell proliferation, alleviate cellular inflammation, reduce ROS production, enhance the cellular antioxidation defense system, and reduce OS-induced damage. Our findings provide the first line of comprehensive evidence that circGLI3 acts as a ceRNA to increase the protein and mRNA expression levels of the target gene VEGFA by competitively binding miR-339-5p.

## Data Availability

The datasets used and/or analyzed during the current study are available from the corresponding author on reasonable request.

## Supplementary Materials

Supplementary Materials Supplementary 1: The fluorescence density analysis of histogram in Figure 3G and 9H. Supplementary 2: the quantification of the ratio of co-localization in Figure 6. Supplementary 3: all original pictures for blot.
