# Supplementary figures and images for: circGLI3 Inhibits Oxidative Stress by Regulating the miR-339-5p/VEGFA Axis in IPEC-J2 Cells
Source: Biomed Res Int. 2021 Aug 11;2021:1086206. doi: 10.1155/2021/1086206 (PMC8376464; doi:10.1155/2021/1086206)

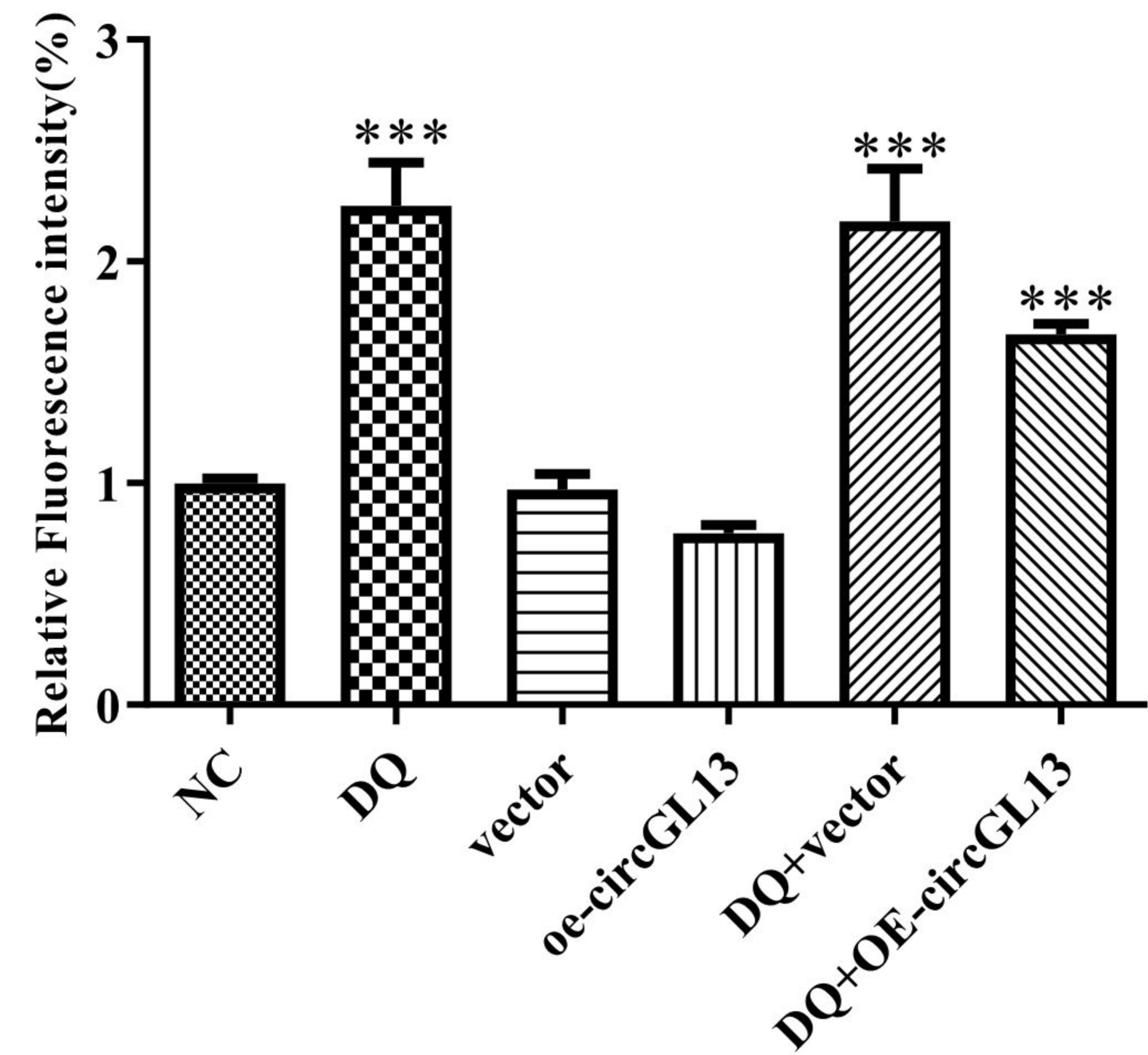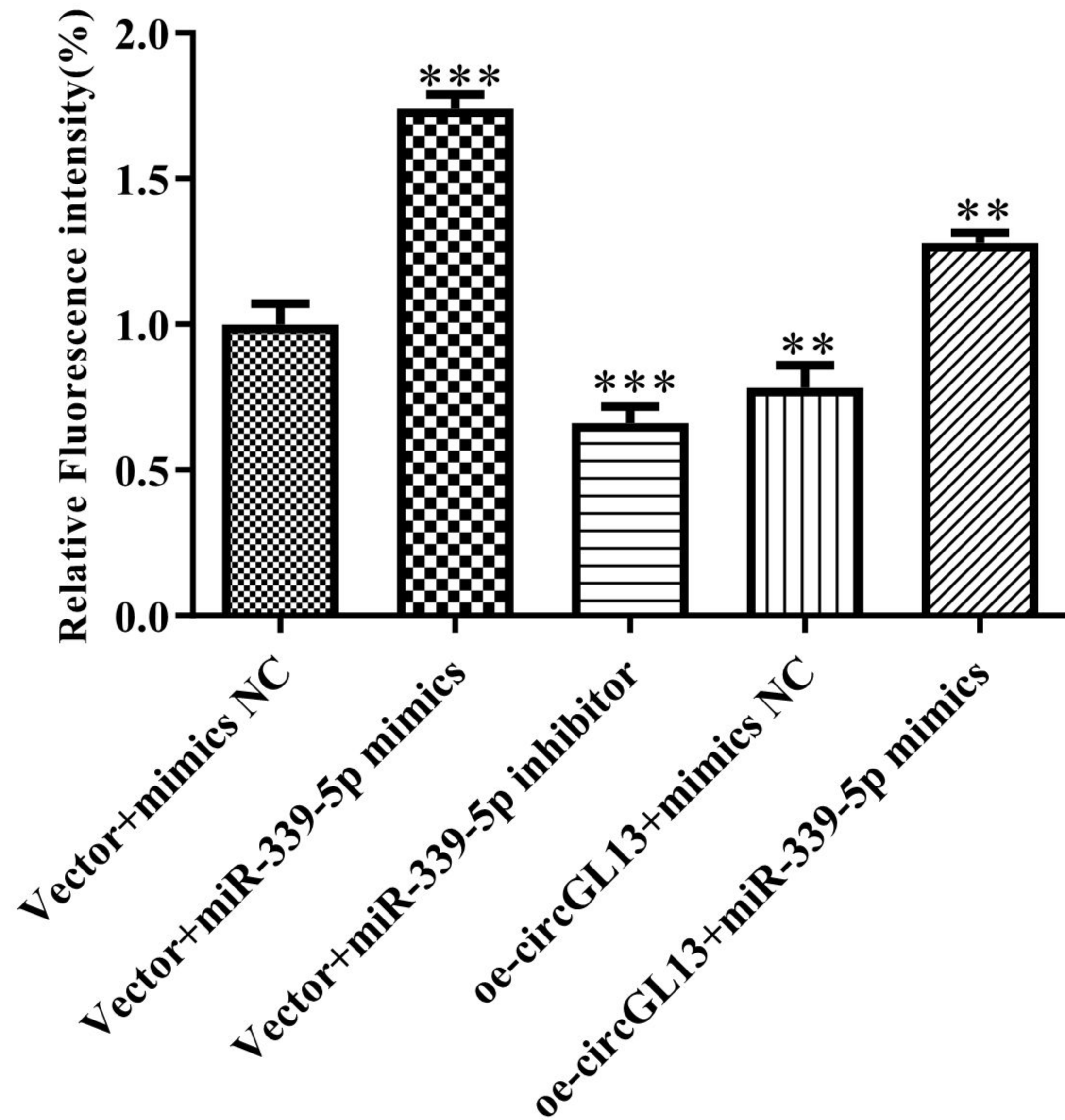

Supplement: Supplementary Materials — Supplementary 1: The fluorescence density analysis of histogram in Figure 3G and 9H. Supplementary 2: the quantification of the ratio of co-localization in Figure 6. Supplementary 3: all original pictures for blot. [file 1086206.f1.zip › 1086206.f1/Supplementary 1.pdf]

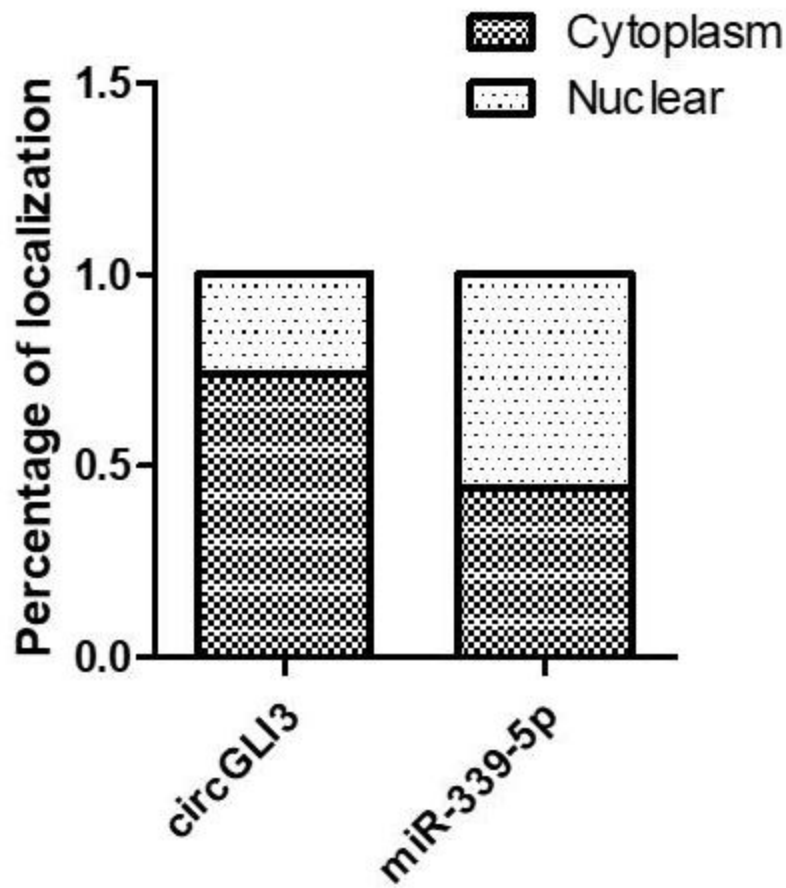

Supplement: Supplementary Materials — Supplementary 1: The fluorescence density analysis of histogram in Figure 3G and 9H. Supplementary 2: the quantification of the ratio of co-localization in Figure 6. Supplementary 3: all original pictures for blot. [file 1086206.f1.zip › 1086206.f1/Supplementary 2.pdf]

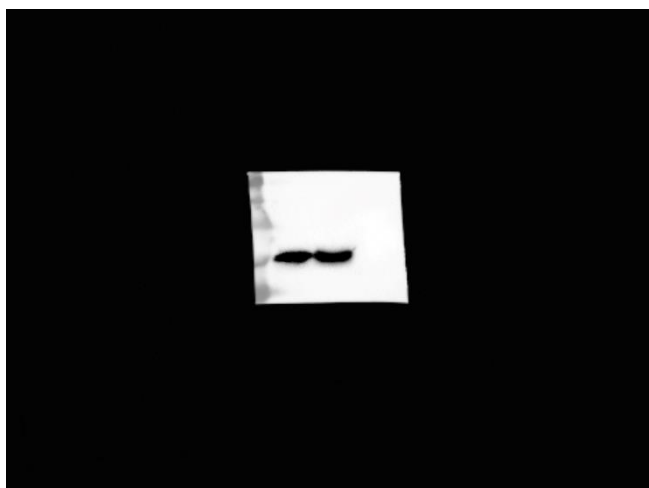

GAPDH (Figure 8C)

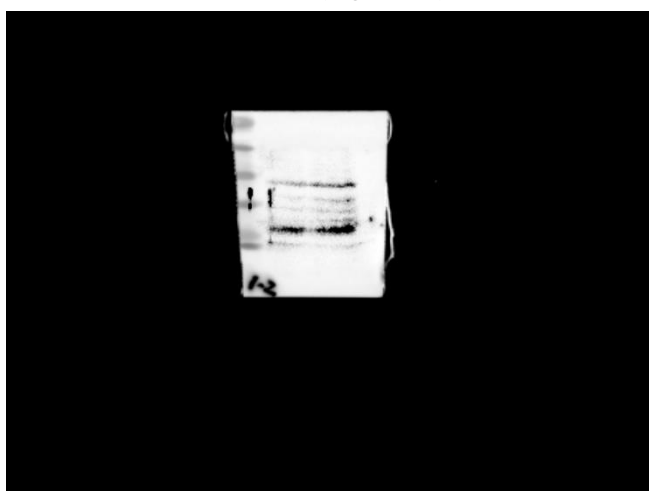

VEGFA (Figure 8C)

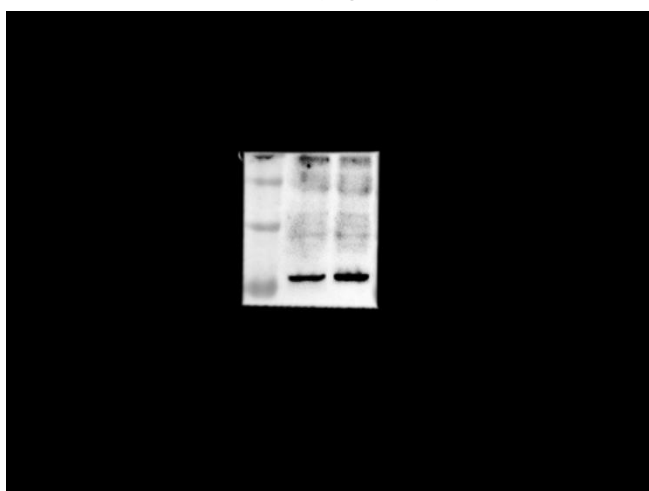

VEGFA (Figure 8C)

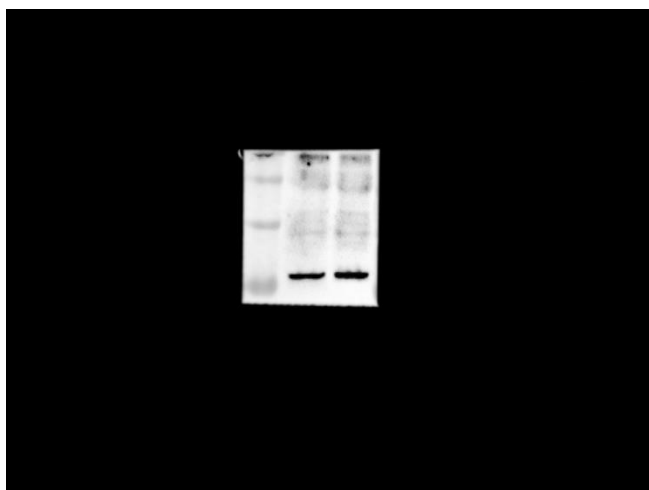

VEGFA (Figure 8C)

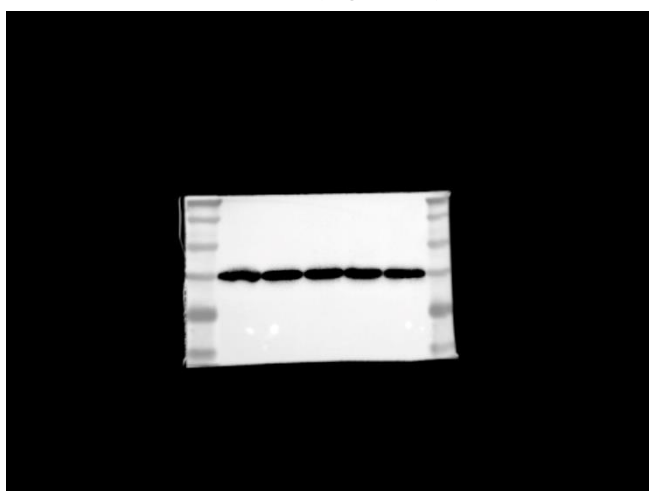

GAPDH (Figure 8D)

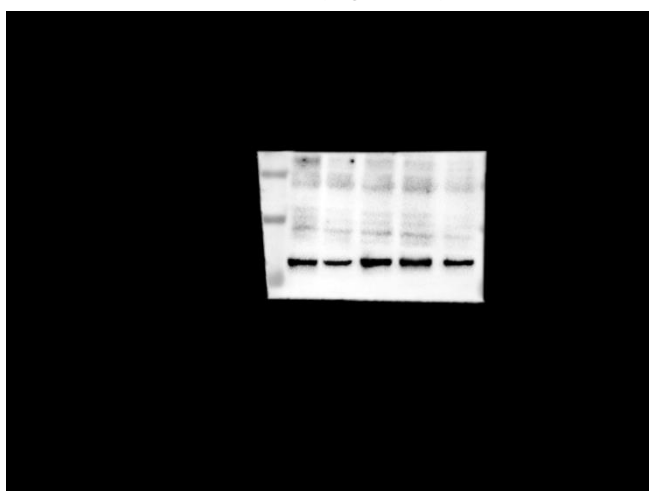

VEGFA (Figure 8D)

Supplement: Supplementary Materials — Supplementary 1: The fluorescence density analysis of histogram in Figure 3G and 9H. Supplementary 2: the quantification of the ratio of co-localization in Figure 6. Supplementary 3: all original pictures for blot. [file 1086206.f1.zip › 1086206.f1/Supplementary 3.pdf]
